# Limited Comparative Evidence Between Metabolic Bariatric Surgery and Incretin‐Based Pharmacotherapy for Weight Loss in Type 2 Diabetes: A Systematic Review and Meta‐Analysis

**DOI:** 10.1111/1753-0407.70259

**Published:** 2026-08-04

**Authors:** Oqab Baniowdeh, Ahmad Arar, Yusuf Muslih, Tala Obeidat, Eliana Marji, Shahed Elghandour, Jalal Jbara, Lela Shengelia

**Affiliations:** ^1^ School of Medicine University of Georgia Tbilisi Georgia; ^2^ Tbilisi State Medical University Tbilisi Georgia

## Abstract

Current comparative evidence between metabolic bariatric surgery and incretin‐based pharmacotherapy for weight loss in adults with type 2 diabetes remains limited. Our systematic review and meta‐analysis suggests greater weight loss following metabolic bariatric surgery while highlighting the urgent need for high‐quality prospective head‐to‐head comparative studies.

## Graphical abstract

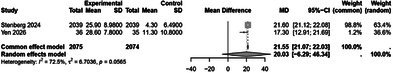

Dear Editor,

We would like to share findings from our recent systematic review and meta‐analysis evaluating the comparative evidence between metabolic bariatric surgery and incretin‐based pharmacotherapy for weight loss in adults with type 2 diabetes mellitus.

The rapid expansion of incretin‐based pharmacotherapy, including glucagon‐like peptide‐1 receptor agonists (GLP‐1 RAs) and newer dual incretin therapies, has transformed the management of obesity and type 2 diabetes mellitus (T2DM) [1, 2, 3]. At the same time, metabolic bariatric surgery remains the most effective intervention for achieving substantial and sustained weight loss [4, 5]. Despite increasing use of both approaches, direct comparative evidence remains limited.

To evaluate the current evidence, we conducted a systematic review and meta‐analysis following PRISMA guidelines and prospectively registered the protocol in PROSPERO (CRD420251229824). PubMed, Scopus, and Web of Science were searched for studies published between January 2021 and January 2026 that directly compared incretin‐based pharmacotherapy with metabolic bariatric surgery in adults with T2DM. Four studies met the eligibility criteria, including three retrospective cohort studies and one prospective non‐randomized comparative study [6, 7, 8, 9].

Two studies reporting outcomes between 12 and 24 months were included in the quantitative synthesis. Random‐effects meta‐analysis suggested greater percentage total body weight loss following metabolic bariatric surgery compared with incretin‐based pharmacotherapy (mean difference 20.03%, 95% confidence interval −6.29 to 46.34), although statistical heterogeneity was substantial (*I* ^2^ = 72.5%) Figure 1. Two additional studies reporting longer‐term follow‐up were synthesized narratively and consistently demonstrated greater and more sustained weight reduction following surgical intervention [6, 7, 8, 9].

**FIGURE 1:**
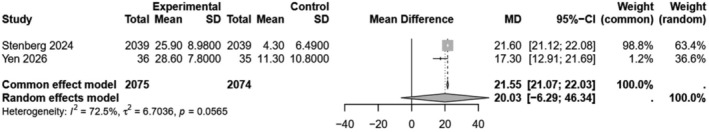
Comparison of percentage total body weight loss (%TWL) between metabolic bariatric surgery and incretin‐based pharmacotherapy in adults with type 2 diabetes mellitus. Forest plot showing pooled mean difference in percentage total body weight loss (%TWL) between treatment groups. Positive values favor metabolic bariatric surgery. Random‐effects model with Hartung–Knapp adjustment was used.

However, interpretation of these findings requires caution. Available studies were predominantly observational and susceptible to residual confounding. In addition, incretin‐based pharmacotherapy encompassed heterogeneous agents with differing pharmacological potency, while variation in bariatric surgical procedures was not consistently reported. Patients selected for metabolic surgery may also differ systematically from those receiving pharmacotherapy with respect to obesity severity, comorbidity burden, and treatment history, limiting direct comparability between treatment groups [6, 7, 8, 9].

The most important finding of our review may therefore be the scarcity of robust comparative evidence rather than the magnitude of the observed treatment effect. While the overall direction of evidence suggests greater weight loss following metabolic bariatric surgery, current data remain insufficient to establish definitive comparative effectiveness between these treatment strategies.

Given the increasing clinical use of both metabolic surgery and newer incretin‐based therapies, there is an urgent need for prospective head‐to‐head studies using standardized outcome measures, comparable follow‐up periods, and rigorous methods to minimize treatment‐selection bias. Such evidence will be essential to inform clinical decision‐making and optimize treatment selection for patients with T2DM [10, 11].

## Funding

The authors have nothing to report.

## Conflicts of Interest

The authors declare no conflicts of interest.

## Data Availability

The data that support the findings of this study are available from the corresponding author upon reasonable request.
